# The association between the PPARγ2 Pro12Ala polymorphism and nephropathy susceptibility in type 2 diabetes: a meta-analysis based on 9,176 subjects

**DOI:** 10.1186/1746-1596-8-118

**Published:** 2013-07-15

**Authors:** Lei Wang, Zan Teng, Shuang Cai, Difei Wang, Xin Zhao, Kai Yu

**Affiliations:** 1 Department of Gerontology and Geriatrics, the First Hospital of China Medical University, NO.155, North Nanjing Street, Heping District, Shenyang 110001 China; 2 Department of Medical Oncology, the First Hospital of China Medical University, NO.155, North Nanjing Street, Heping District, Shenyang 110001 China; 3 Department of Pharmacy, the First Hospital of China Medical University, NO.155, North Nanjing Street, Heping District, Shenyang 110001, China; 4 Department of Endocrinology and Metabolism, the First Hospital of China Medical University, NO.155, North Nanjing Street, Heping District, Shenyang 110001, China

**Keywords:** PPARγ2, Meta-analysis, Diabetic nephropathy, Polymorphisms

## Abstract

**Background:**

The polymorphism Pro12Ala in peroxisome proliferator-activated receptor­γ2 gene (PPARγ2) has been reported to be associated with diabetic nephropathy (DN) in some studies, though the results remain inconclusive. To explore this relationship between PPARγ2 Pro12Ala polymorphism and the susceptibility for DN, a cumulative meta-analysis was performed in this study.

**Method:**

PubMed, Medline, Embase and Web of Science databases have been systematically searched to identify relevant studies. Odds ratios (ORs) and 95% confidence intervals (CIs) were calculated.

**Results:**

18 studies were included in this meta-analysis, involving 3,361 cases and 5,815 controls. The PPARγ2 Ala12 allele was significantly associated with decreased risk of DN based on dominant model (OR=0.778; 95%CI=0.618–0.981; P_heterogeneity_=0.008; P=0.034). In the stratified analysis by ethnicity, significantly decreased risks were found among Caucasians for dominant model (OR=0.674; 95%CI=0.500–0.909; P_heterogeneity_=0.079; P=0.010), while there was no significant association was found in Asians.

**Conclusions:**

The results from the present meta-analysis indicated that the Pro12Ala polymorphism in PPARγ2 gene is not a risk factor for DN in type 2 diabetes (T2D). Further large and well-designed studies are needed to confirm this conclusion.

**Virtual slides:**

The virtual slides for this article can be found here: http://www.diagnosticpathology.diagnomx.eu/vs/7491348341027320.

## Introduction

Diabetic nephropathy is the leading cause of end-stage renal disease (ESRD) worldwide, and it is estimated that 20% of T2D patients reach ESRD during their lifetime [1]. The pathogenesis of DN has many genetic and environmental factors contributing to its development and progression. The risk of developing DN has been linked to different chromosomes, including chromosome 3, to which the peroxisome proliferators-activated receptor (PPAR) gene has been mapped, particularly to the PPAR gamma (PPARG) nuclear receptor, which is mainly expressed in adipose tissue but is also found in pancreatic beta cells, vascular endothelium, and macrophage [2,3].

PPAR-γ2 is a nuclear receptor that serves important roles in intermediate metabolism. The PPARG gene is located on chromosome 3p [2]. The Pro12Ala polymorphism of the PPARG gene, a Pro-to-Ala exchange that results in the substitution of proline with alanine at codon 12, was associated with reductions in both DNA binding and transcriptional activity in vitro, and Ala12 carriers showed significant improvement in insulin sensitivity [4,5]. The Pro12Ala polymorphism in the PPARG gene is suggested to be associated with DN. As we all kwon, many epidemiologic studies investigated the potential association of PPARG gene polymorphisms with susceptibility to DN. However, these studies have reported inconclusive results.

One putative genetic determinant of DN is the Pro12Ala polymorphism in the gene encoding PPARg. PPARg, a member of the nuclear hormone receptor superfamily of ligand-activated transcription factors, plays a key role in regulating the expression of numerous genes involved in lipid metabolism, metabolic syndrome, inflammation, and atherosclerosis [6,7]. The P12A single-nucleotide polymorphism (SNP) located in the adipocyte-specific PPARg2 isoform has been associated with lower nephropathy in T2D [8,9]. The underlying molecular mechanism of this polymorphism appears to be, in vitro, a moderate reduction in DNA-binding activity and reduced transcriptional activity [4,10]. In the present study, we examined the relationship between PPAR-γ2 gene polymorphisms and the risk of T2D DN.

## Materials and methods

### Search strategy

A comprehensive literature search was performed using the PubMed, Medline, Embase, and Web of Science database for relevant articles published (last search updated in May. 2013) with the following key words “PPARγ2”, “polymorphism” and “diabetic nephropathy”. Additional studies were identified by hand searching references in original articles and review articles. Authors were contacted directly regarding crucial data not reported in original articles. The search was limited to human studies. All eligible studies were retrieved, and their bibliographies were checked for other relevant publications. When the same sample was used in several publications, only the most complete study was included following careful examination.

### Inclusion criteria

The included studies have to meet the following criteria: (1) only the case–control studies were considered; (2) evaluated the PPARγ2 Pro12Ala polymorphism and DN risk; (3) the genotype distribution of the polymorphism in cases and controls were described in details, and the results were expressed as odds ratio (OR) and corresponding 95% confidence interval (95% CI).

### Exclusion criteria

Major reasons for exclusion of studies were as follows: (1) not for DN research; (2) only case population; (3) duplicate of previous publication; and (4) the distribution of genotypes among controls are not in Hardy–Weinberg equilibrium (P <0.01).

### Data extraction

Information was carefully extracted from all eligible studies independently by two investigators according to the inclusion criteria listed above. The following data were collected from each study: first author’s name, year of publication, country of origin, ethnicity, source of controls, genotyping method, match, sample size, and numbers of cases and controls in the PPARγ2 Pro12Ala genotypes whenever possible. Ethnicity was categorized as “Caucasian” and “Asian”. When a study did not state which ethnic groups were included or if it was impossible to separate participants according to phenotype, the sample was termed as “mixed population”. We did not define any minimum number of patients to include in this meta-analysis. Articles that reported different ethnic groups and different countries or locations, we considered them different study samples for each category cited above.

### Statistical analysis

Crude odds ratios (ORs) together with their corresponding 95% CIs were used to assess the strength of association between the PPARγ2 Pro12Ala polymorphism and DN risk. The pooled ORs were performed for dominant comparison model (Ala-carrier vs Pro/Pro). Between-study heterogeneity was assessed by calculating Q-statistic (Heterogeneity was considered statistically significant if P < 0.10) [11] and quantified using the *I*^*2*^ value, Venice criteria [12] for the *I*^*2*^ test included: “*I*^*2*^< 25% represents no heterogeneity, *I*^*2*^= 25–50% represents moderate heterogeneity, *I*^*2*^= 50–75% represents large heterogeneity, and *I*^*2*^ >75% represents extreme heterogeneity”. If results were not heterogeneous, the pooled ORs were calculated by the fixed-effect model (we used the Q-statistic, which represents the magnitude of heterogeneity between-studies) [13]. Otherwise, a random-effect model was used (when the heterogeneity between-studies were significant) [14]. We also performed subgroup analysis by ethnicity (Caucasian and Asian). Moreover, sensitivity analysis was performed by excluding a single study each time. In addition, we also ranked studies according to sample size, and then repeated this meta-analysis. Begg’s funnel plots [15] and Egger’s linear regression test [16] were used to assess publication bias. All of the calculations were performed using STATA version 12.0 (STATA Corporation, College Station, TX).

## Results

### Study characteristics

Studies relevant to the searching words were retrieved originally. 18 publications addressing the association between PPARγ2 Pro12Ala polymorphism and DN risk were preliminarily eligible [8,9,17-30]. Table 1 presents the main characteristics of these studies. There were a total of 18 studies including 8 groups of Caucasians and 10 groups of Asians. Given the low frequency of Ala allele, even in some studies, Ala homozygous individuals are absent, and only the dominant model was investigated, comparing Ala carriers to Pro/Pro. We also assessed the deviation of Hardy-Weinberg equilibrium in control subjects, and the results demonstrated that all the distribution of genotypes in the controls of all studies was in agreement with Hardy–Weinberg equilibrium.

**Table 1:** Main characteristics of these studies included in this meta-analysis

**First author**	**Year**	**Country**	**Ethnicity**	**Genotype method**	**Cases**		**Controls**		***HWE***
					**Pro/ Pro**	**Ala-carrier**	**Pro/ Pro**	**Ala-carrier**	
Mori et al.	2001	Japanese	Asian	PCR-RFLP	580	28	982	42	Yes
Herrmann et al.	2002	German	Caucasian	PCR-RFLP	154	43	144	59	Yes
Caramori et al.	2003	Brazilian	Caucasian	PCR-RFLP	93	11	169	43	Yes
Wu et al.	2004	Chinese	Asian	PCR-RFLP	194	26	102	6	Yes
Maeda et al.	2004	Japanese	Asian	PCR-RFLP	46	15	55	24	Yes
Pollex et al.	2007	Canadian	Caucasian	PCR-RFLP	94	3	55	7	Yes
Erdogan et al.	2007	Turk	Asian	PCR-RFLP	43	0	47	1	Yes
Wei et al.	2008	Chinese	Asian	PCR-RFLP	68	14	89	10	Yes
Li et al.	2008	Chinese	Asian	PCR	150	15	77	17	Yes
Wu et al.	2009	Taiwanese	Asian	Taqman	157	18	197	17	Yes
De Cosmo et al.	2009	Italian	Caucasian	Taqman	86	7	856	170	Yes
Liu et al.	2010	Chinese	Asian	PCR-RFLP	499	33	199	29	Yes
Lapice et al.	2010	Italian	Caucasian	PCR-RFLP	53	2	606	89	Yes
Zhu et al.	2011	Chinese	Asian	PCR-RFLP	39	2	33	4	Yes
De Cosmo et al^1^	2011	Italian	Caucasian	SBE	221	40	499	81	Yes
De Cosmo et al^2^	2011	Italian	Caucasian	Taqman	224	30	316	53	Yes
De Cosmo et al^3^	2011	Italian	Caucasian	ASA	207	25	422	60	Yes
Zhang et al.	2012	Indian	Asian	PCR-RFLP	113	28	206	49	Yes

**PCR-RFLP**: Polymerase Chain Reaction-Restriction Fragment Length Polymorphism; **PCR**: Polymerase Chain Reaction; **SBE**: Single-Base Extension; **ASA**: Allele-Specific Amplification; **1,2,3**: Different studies in one publication; ***HWE***: Hardy–Weinberg Equilibrium; **Yes**: **P**_***HWE***_ value>0.05.Reaction; **SBE**: Single-Base Extension; **ASA**: Allele-Specific Amplification; **1,2,3**: Different studies in one publication; ***HWE***: Hardy–Weinberg Equilibrium; **Yes**: **P**_***HWE***_ value>0.05.

### Meta-analysis results

**Figure 1:**
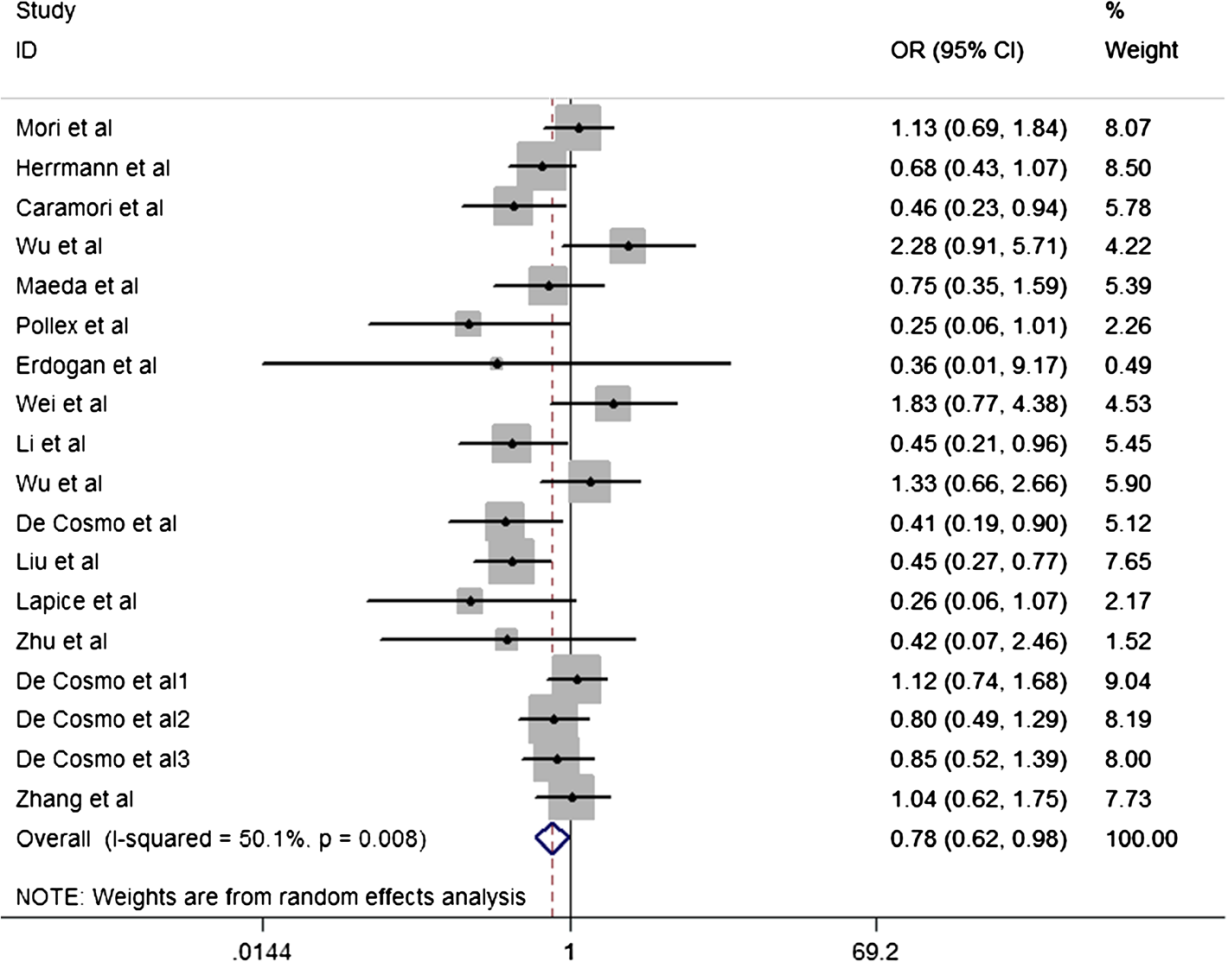
Forest plots of the meta-analysis for the association between PPARγ2 Pro12Ala and nephropathy in type 2 diabetes patients.

**Figure 2:**
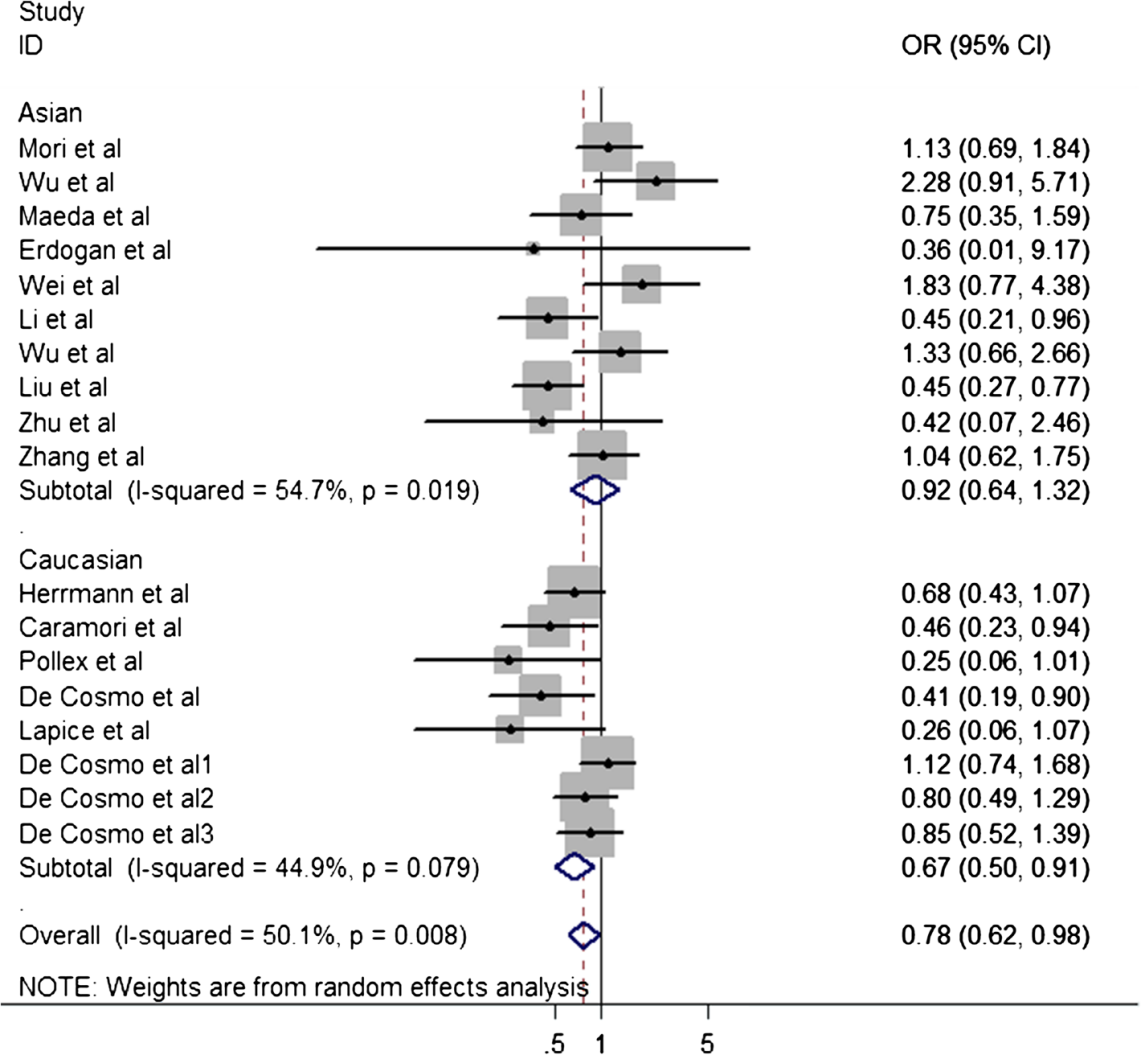
Subgroup meta-analysis was held by ethnicity for the association between PPARγ2 Pro12Ala and nephropathy in type 2 diabetes patients.

Overall, the Pro12Ala polymorphism was found to be significantly associated with decreased risk of T2D DN (OR=0.778; 95%CI=0.618–0.981; P_heterogeneity_=0.008; P=0.034) (Figure 1). Both the Cochran Q test and estimate of *I*^*2*^ revealed significant heterogeneity among the constituent studies (P_heterogeneity_=0.008; *I*^*2*^=50.1%). To avoid the influence of heterogeneity among the included studies, subgroup analyses were distinctively carried out according to the ethnicity. In the stratified analysis by ethnicity, significantly decreased risks were found among Caucasians for dominant model (Ala carrier vs Pro/Pro: OR=0.674; 95%CI=0.500–0.909; P_heterogeneity_=0.079; P=0.010), while there was no significant association was found under the same genetic model (OR=0.917; 95%CI=0.639-1.315; P_heterogeneity_=0.019; P=0.637) (Figure 2). Significant heterogeneity was found in Asian populations (P_heterogeneity_=0.019; *I*^*2*^= 54.7%), but not found in Caucasian populations (P_heterogeneity_=0.079; *I*^*2*^=44.9%).

### Sensitivity analysis

**Figure 3:**
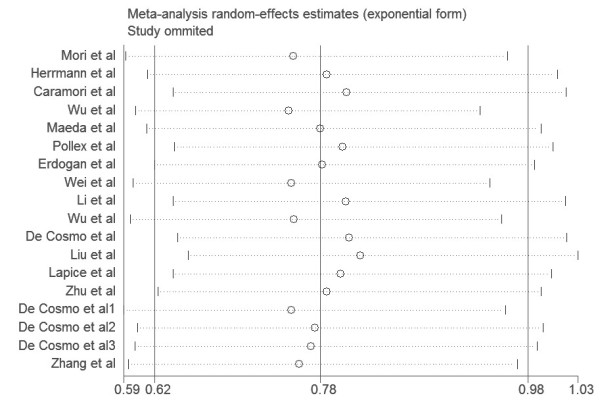
One-way sensitivity analysis of the pooled ORs and 95% CI for the overall analysis, omitting each dataset in the meta-analysis.

To test the stability of the pooled results, one-way sensitivity analyses were performed. A single study involved in the meta-analysis was deleted each time to reflect the influence of the individual data set to the pooled ORs, no other single study influenced the pooled OR qualitatively, suggesting that the results of this meta-analysis were stable (Figure 3).

### Publication bias

**Figure 4:**
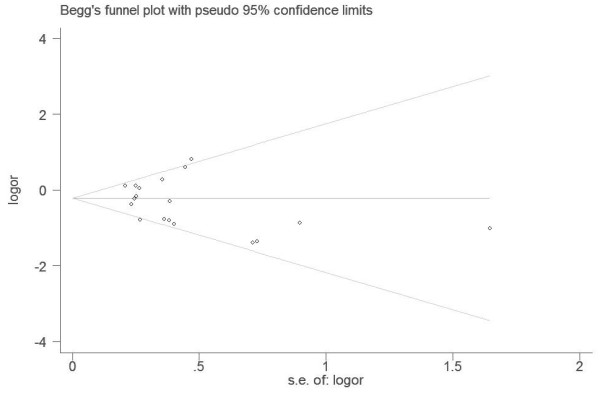
Begg funnel plot analysis to detect potential publication bias.

**Figure 5:**
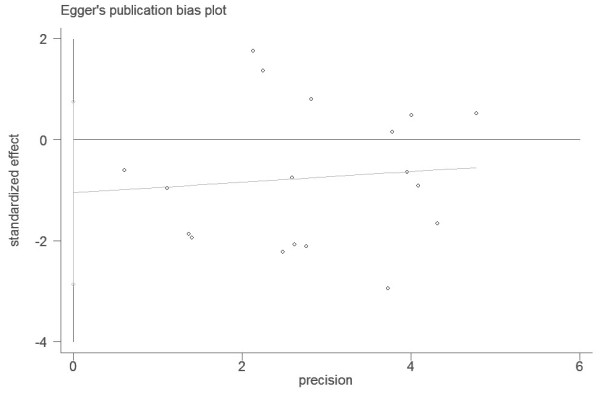
Egger’s test was held to detect potential publication bias.

Begg’s funnel plot and Egger’s test were performed to assess the publication bias of the literature. The shapes of the funnel plots did not reveal any evidence of obvious asymmetry (P_Begg_=0.472) (Figure 4). Furthermore, Egger’s test was used to provide statistical evidence for funnel plot symmetry. The results still did not suggest any evidence of publication bias (P_Eegger_=0.234) (Figure 5).

## Discussion

It is well recognized that there is individual susceptibility to the DN even with the same environmental exposure. Host factors, including polymorphisms of genes involved in this formation of DN may have accounted for this difference. Therefore, genetic susceptibility to DN has been a research focus in scientific community. Recently, genetic polymorphisms of the PPARγ2 Pro12Ala gene in the etiology of DN have drawn increasing attention. Previous results of the studies on the relationship between PPARγ2 Pro12Ala polymorphisms and DN risk were contradictory. These inconsistent results are possibly because of a small effect of the polymorphism on DN risk or the relatively low statistical power of the published studies. A meta-analysis is a powerful strategy because it potentially investigates a large number of individuals and can estimate the effect of a genetic factor on the risk of the disease [31-35]. To better understanding of this association, a pooled analysis with a large sample, subgroup analysis performed, and heterogeneity explored is necessary to provide a quantitative approach for combining the results of various studies with the same topic, and for estimating the real association in this meta-analysis.

The present study including 3,361 cases and 5,815 controls from 18 published case–control studies, explored the association between a potentially functional polymorphism, PPARγ2 Pro12Ala and T2D DN risk. We found that there was evidence that the variant genotypes of the PPARγ2 were associated with a significant decreased overall risk of DN. When stratified by ethnicity, Caucasians with the Ala carrier showed a decreased risk of DN compared with those with the Pro/Pro genotype. However, Asians did not show the same results.

Some limitations of this meta-analysis should be mentioned. Firstly, bladder cancer is a multifactorial disease that results from complex interactions between many environmental and genetic factors. This means that there will not be single gene or single environmental factor that has large effects on DN susceptibility. Our results were based on unadjusted estimates, while a more precise analysis should be conducted if individual data were available, which would allow for the adjustment by other covariates including age, sex, family history, environmental factors and lifestyle. Secondly, the number of subjects was relatively small, not having enough statistical power to explore the real association. Thirdly, the controls were not uniformly defined. Finally, there were only two ethnic decent populations. So, further large and well-designed studies are needed to confirm this conclusion.

## Conclusion

This meta-analysis suggests that the PPARγ2 Pro12Ala polymorphism is not a risk factor for developing T2D DN. Moreover, gene–gene and gene–environment interactions should also be considered in future analysis. Such studies taking these factors into account may eventually lead to our better, comprehensive understanding of the association between the PPARγ2 Pro12Ala polymorphism and T2D DN risk.

## Abbreviations

DN: Diabetic nephropathy; HWE: Hardy–Weinberg equilibrium; OR: Odds ratio; CI: Confidence interval; PPARγ2: Peroxisome proliferator-activated receptor­γ2 gene.

## Competing interests

Both authors declared that they have no conflict interest in relation to this study.

## Authors’ contributions

Lei Wang, Zan Teng, Shuang Cai,Difei Wang, Xin Zhao and Kai Yu drafted the manuscript, and carried out the molecular genetic studies, participated in the sequence alignment, Lei Wang reviewed the manuscript finally. All authors read and approved the final manuscript.
